# Molecular and Clinical Aspects of COVID-19 Vaccines and Other Therapeutic Interventions Apropos Emerging Variants of Concern

**DOI:** 10.3389/fphar.2021.778219

**Published:** 2021-12-23

**Authors:** Khursheed Ul Islam, Thoraya Mohamed Elhassan A-Elgadir, Sarah Afaq, Tanveer Ahmad, Jawed Iqbal

**Affiliations:** ^1^ Multidisciplinary Centre for Advanced Research and Studies, Jamia Millia Islamia, New Delhi, India; ^2^ Department of Clinical Biochemistry, College of Medicine, King Khalid University, Abha, Saudi Arabia

**Keywords:** SARS-CoV-2, COVID-19, vaccines, variant of concern, therapeutics

## Abstract

Coronavirus disease 2019 (COVID-19) has overwhelmed the healthcare and economy of the world, with emerging new variants of severe acute respiratory syndrome coronavirus 2 (SARS-CoV-2) posing an everlasting threat to humanity. While most COVID-19 vaccines provide adequate protective immunological response against the original SARS-CoV-2 variant, there is a pressing need to understand their biological and clinical responses. Recent evidence suggests that some of the new variants of SARS-CoV-2 evade the protection conferred by the existing vaccines, which may impede the ongoing efforts to expedite the vaccination programs worldwide. These concerns have also highlighted the importance of a pan-COVID-19 vaccine, which is currently in the making. Thus, it is imperative to have a better molecular and clinical understanding of the various COVID-19 vaccines and their immunological trajectory against any emerging variant of concerns (VOCs) in particular to break this vicious cycle. Furthermore, other treatment regimens based on cellular therapies and monoclonal antibodies should be explored systematically as an alternative and readily available option considering the possibility of the emergence of more virulent SARS-CoV-2 mutants. In this review, we shed light on the various molecular mechanisms and clinical responses of COVID-19 vaccines. Importantly, we review the recent findings of their long-term immune protection and efficacy against emerging VOCs. Considering that other targeted and effective treatments will complement vaccine therapy, we provide a comprehensive understanding of the role of cell-based therapies, monoclonal antibodies, and immunomodulatory agents as alternative and readily available treatment modalities against any emerging SARS-CoV-2 variant.

## Introduction

Severe acute respiratory syndrome coronavirus-2 (SARS-CoV-2) emerged as a new virus from a wild market in Wuhan City, China, and had spread rapidly to almost every part of the world (Zheng 2020). Due to its highly contagious nature, the SARS-CoV-2 outbreak was declared as pandemic on March 11, 2020 by the World Health Organization (WHO) (Cucinotta and Vanelli 2020). SARS-CoV-2 causes a coronavirus disease called COVID-19 that has resulted in more than 4.87 million deaths worldwide as per the WHO report on October 15, 2021 (https://covid19.who.int/). The COVID-19 outbreak and its widespread disruption in health and the economy have prompted scientists to look for different therapeutic strategies to minimize viral spread and the morbidities associated with it. Different therapeutic approaches such as convalescent therapy, monoclonal antibody treatment, immunotherapies, cell-based therapies, and vaccine therapeutics are available and used to combat SARS-CoV-2 infection (Ali et al., 2020). Passive immunotherapy in the form of convalescent therapy has been used since the late 19th century against infectious diseases, recently reviewed by Katz (2021). Convalescent plasma has also shown moderate success in a subset of COVID-19 patients, while in others this treatment offered very little respite (Nagoba et al., 2020).

Owing to the novelty of SARS-CoV-2 and its rapid spread, it was a challenging task to develop a new drug or a therapeutic agent at the earliest. In this challenging situation, some repurposed drugs and later immunotherapeutic options were introduced to limit hospitalizations due to COVID-19. The immunotherapeutic approaches were based on recombinant proteins or monoclonal antibodies generated against various antigens of SARS-CoV-2 (Wang et al., 2020). These antibody-based therapies became available as intravenous injection and, in some cases, as easy-to-use nasal sprays (Kwon 2021; Taylor et al., 2021). However, the major breakthrough treatment came in the form of the much anticipated COVID-19 vaccines (Kyriakidis et al., 2021). History tells us that developing a vaccine against a novel pathogen is a time-consuming process. However, the early development of the COVID-19 vaccines took the whole world by surprise, and the scientific community as well, as the general public had apprehensions about the safety, efficacy, and long-term health impact of these vaccines. About 126 COVID-19 vaccine candidates are currently in various stages of clinical development and around 194 in the preclinical stage according to the WHO report in August 2021 (https://www.who.int/publications/m/item/draft-landscape-of-covid-19-candidate-vaccines).

Now, as large populations worldwide are getting vaccinated, we have data regarding the safety, efficacy, and immunological response of these vaccines and other therapeutic interventions. Thus, in this review, we will discuss the molecular and clinical responses of various immunotherapeutic modalities, specifically the COVID-19 vaccine-based therapeutic interventions that are currently used to limit SARS-CoV-2 infection and treat COVID-19.

## Immunomodulatory-Based Therapeutic Approaches for COVID-19

Considering the complexity of the immune response developed due to COVID-19, it is imperative to contemplate on the underlying immune response while designing the best possible therapeutic interventions (Roberts et al., 2020). As most of the complications seem to arise due to hyperactivated immune response-associated acute respiratory distress syndrome (ARDS), drugs modulating the pro-inflammatory responses may be effective (Gibson and Qin, 2020). In this regard, immunotherapies and cell-based therapies are emerging as the promising therapeutic modality for the treatment of severe and critically ill patients (AminJafari and Ghasemi 2020). Immunotherapies targeting the cytokines implicated as the hallmark in cytokine storm and adoptive cell-based therapies such as mesenchymal stem cells (MSCs), T cells, and natural killer (NK) cell-based therapies are in various phases of clinical testing for the treatment of COVID-19 (Saeedi and Halabian, 2019; Musial and Gorska-Ponikowska 2021).

### Convalescent Plasma Therapy

An immediate and readily available source to treat severe and critically ill COVID-19 patients is by taking advantage of traditional convalescent plasma therapy (CPT). The presence of virus-specific antibodies has been detected in most of the patients who have successfully eliminated the virus (Ahmad et al., 2020; Casadevall and Pirofski 2020). The plasma containing antibodies from the recovered patients are thus given to the infected patients who could not mount an adequate humoral response (Luke et al., 2006). This traditional approach is based on the principle that the neutralizing antibodies from the recovered patients will provide a similar and durable response in the infected patients who receive the CPT. Antibodies will either neutralize the virus or enhance their uptake by phagocytosis. Additionally, these antibodies will also activate other effector immune cells to eliminate the virus-infected cells by activating other immune functions such as NK cells. Considering the high success rate of CPT in previous SARS-CoV-1-infected patients (Cheng et al., 2005; Yeh et al., 2005), it is plausible to use a similar approach for COVID-19. The ease of availability of plasma from a large number of infected COVID-19 donors seems a rational therapeutic option at hand before any other potential therapies become available for emerging variants of concern (VOCs), which escape the immunity conferred by the existing vaccines (Lopez Bernal et al., 2021). Previous data from SARS-CoV-2-infected individuals treated with CPT showed moderate success in a subset of patients. In a small cohort of 10 severe cases of COVID-19, Duan et al. showed that all patients successfully recovered after convalescent plasma transfusion (Duan et al., 2020). Similarly, the study by Shen et al. demonstrated 100% recovery in five critically ill patients after they received the plasma transfusion (Shen et al., 2020). Furthermore, in a case study on a single critically ill patient who exhibited respiratory distress, CPT along with remdesivir showed a promising recovery rate (Anderson et al., 2020). Many other early clinical reports have shown promising results with CPT, reviewed by Casadevall et al. (2020). Besides, more than 100 clinical trials are registered in ClinicalTrials.gov.in. These early clinical findings thus suggest that CPT is a potential therapeutic intervention for severe and critically ill patients and should be explored in cases of emergent new variants, where vaccines are not available or have failed to provide protection (Kanj and Al-Omari 2021). However, caution should be exercised while using this approach as many studies have shown contradicting results (Zhao and He 2020; Tiberghien et al., 2020). Particularly, to ensure virus-free plasma, optimization of the dosage and time of delivery should be critically evaluated (Duan et al., 2020).

### Targeted Immunotherapy Approaches for COVID-19

Consistently, the levels of interleukin 6 (IL-6) are increased in severe and critically ill COVID-19 patients, which is thus being considered as the prognostic and predictive disease biomarker along with the numbers of neutrophil/lymphocytes (Liu et al., 2020; Santa Cruz, 2021). The presence of increased levels of IL-6 are detected in blood and in the bronchoalveolar lavage fluid (BALF), thus making this cytokine as a potential therapeutic target. Approaches to neutralize IL-6 usually rely on the use of anti-IL6R antibody to inhibit IL-6 signaling. Previous studies on cytokine release syndrome (CRS) during cancer immunotherapy have shown clinical success of the anti-IL6R-directed antibody tocilizumab (TCZ) (Khadka et al., 2019). Furthermore, TCZ is an approved drug for the treatment of other inflammatory conditions such as rheumatoid arthritis (Yazici et al., 2012). Thus, it was rational to test this drug for the treatment of COVID-19. Several reports have suggested the beneficial effect of TCZ in severe and critical COVID-19 patients. The study by Luo et al. found that out of 15 patients, 11 responded well to the treatment and showed stable disease condition and a marked decrease in C-reactive protein (CRP) levels (Luo et al., 2020).

In a cohort of 100 critically ill patients, TCZ treatment was associated with a high recovery rate and a significant improvement in cytokine storm and clinical symptoms (Toniati et al., 2020) Similarly, a 92% recovery rate was observed in patients who received TCZ treatment in comparison to the 42.1% recovery rate in patients who received a combination therapy of hydroxychloroquine, lopinavir, and ritonavir (Chen et al., 2020). Surprisingly, in a very recent study by Xu et al., a 100% recovery rate was observed in 20 severe and critically ill patients who received TCZ treatment (Xu et al., 2020). Similarly, others have also observed the beneficial effect of TCZ (Luo et al., 2020). On the contrary, TCZ was shown to exhibit no added benefit in intensive care unit (ICU) admissions or mortality rate in a cohort of 21 patients who were compared with a combination therapy of hydroxychloroquine, azithromycin, and a prophylactic dose of low-molecular-weight heparin (Colaneri et al., 2020). Similarly, the study carried out by Carlo Salvarani et al. compared TCZ treatment with standard care on the clinical worsening of COVID-19 patients. In this study, it was found that clinical worsening in COVID-19 patients was not effectively prevented by the administration of TCZ (Salvarani et al., 2021). The probable reason for this discrepancy could be the dose, time, and the combinatorial effect of other drugs, which were given along with TCZ. Furthermore, the underlying health conditions and comorbidities may have an impact on the therapeutic outcome of this treatment, which needs further exploration. Overall, the results with TCZ have been mostly promising so far, but its use in combination with other antiviral and anti-inflammatory drugs has shown better results (Zhao et al., 2021). Currently, there are more than 50 clinical trials registered in ClinicalTrials.gov evaluating the potential efficacy of TCZ to minimize the cytokine storm and associated immunopathology. These studies will provide a detailed account of the therapeutic efficacy of TCZ for the treatment of COVID-19.

Approaches handling angiogenesis such as anti-VEGF (vascular endothelial growth factor) antibody (bevacizumab) or complement activation inhibitors such as anti-C5a antibody (eculizumab and avdoralimab) are also being explored **(** Billioti de Gage et al., 2021). Other immunotherapy approaches that were investigated include blocking the inhibitory T-cell receptors by anti-PD-1 (programmed cell death 1) or anti-CTLA4 (cytotoxic T-lymphocyte-associated protein 4) antibody (Pardoll and Drew, 2012). These approaches have been successfully used in cancer immunotherapy and other viral infections (Velu et al., 2009). Similarly, immunotherapeutic approaches to handle cytokine storm are in various phases of clinical trials or already approved for emergency use, such as therapies based on anti-IL-6, anti-GM-CSF (granulocyte–macrophage colony-stimulating factor), anti-IL-1β, anti-JAK (Janus kinase), anti-CCR5, anti-TIGIT [T-cell immunoreceptor with immunoglobulin (Ig) and immunoreceptor tyrosine-base inhibitory motif (ITIM) domains], and anti-IFN-γ (Buckley et al., 2020). Thus, exploring the use of these targeted therapies for emergency use seems a rational and feasible approach, considering that T-cell exhaustion is highly prevalent in COVID-19 (De Biasi et al., 2020).

### Mesenchymal Stem Cell-Based Therapy for COVID-19

Owing to their immunomodulatory activity, MSCs exhibit greater potential to modulate the immunological response. Over the years, MSCs have shown greater promise for the treatment of chronic lung disease such as asthma, chronic obstructive pulmonary disease (COPD), pulmonary fibrosis, and acute lung injury (Harrell et al., 2019). The therapeutic potential of these cells is largely exhibited by their paracrine secretion of a range of anti-inflammatory molecules. Our own work has also established the therapeutic efficacy of MSCs in preclinical animal models of various lung diseases (Ahmad et al., 2014). MSCs have been used for the treatment of acute lung diseases such as ARDS, as reviewed by Rogers et al. (2020). Also, early studies in ARDS models have also strengthened this notion that MSCs have an impressive potential to be used as a therapeutic approach to manage the consequences of a hyperactive immune system (Mei et al., 2010). Previous studies have established the safety and efficacy of these cells in patients with ARDS (Simonson, 2015; Wilson et al., 2015; Matthay et al., 2019). Thus, owing to their safety profile and immunomodulatory activity, MSCs can be tested as a viable therapeutic option for the treatment of hyper-inflammatory state in COVID-19 patients. Clinical studies have suggested the early efficacy of these cells in COVID-19 patients (Wang et al., 2021). Last year, a 65-year-old COVID-19 patient showed recovery from ICU in about a week after treatment with umbilical cord-derived MSCs. In another study of seven patients, MSCs resulted in the improvement of disease after just 2 days of treatment. MSC treatment was associated with a significant decline in TNF-α and CRP, with a concomitant increase in anti-inflammatory IL-10 levels (Leng et al., 2020). Besides their direct immunomodulatory potential, MSC-derived products such as exosomes also hold great therapeutic promise. Sengupta et al. evaluated the effect of exosomes derived from allogeneic bone marrow MSCs. Interestingly, these immunomodulatory molecules showed 71% recovery rate in a cohort of 24 severe and critically ill patients (Sengupta et al. 2020). Thus, it is plausible that MSCs may hold promise in alleviating the hyper-inflammatory state in COVID-19 patients infected with new variants of SARS-CoV-2. Several ongoing clinical trials are currently underway to explore in a holistic manner the protective effects of MSCs against COVID-19 (Leng et al., 2020; Moll et al., 2020; Saleh et al., 2021).

### Other Adoptive Cell Transfer-Based Therapies for COVID-19

Adoptive cell transfer (ACT) is emerging as the most promising approach for the treatment of cancer. The current approach uses chimeric antigen-expressing T cells called CAR-T cells for the treatment of hematological malignancies, reviewed by Landoni and Savoldo (2018). The overwhelming success rate of this approach has encouraged researchers to use a similar approach for the treatment of COVID-19. However, limitations associated with this approach may hamper its use in COVID-19, specifically the associated high cost and CRS, which may additionally contribute to hyper-inflammatory response. Besides, CAR-T cell therapy has mostly remained confined to autologous approaches, while it is difficult to obtain the optimal functional T cells from severe and critically ill COVID-19 patients due to lymphopenia and lymphocyte exhaustion (Zheng et al., 2020). Thus, an alternative approach based on NK cells is currently being investigated, as NK cell-based therapy is not associated with CRS and can be used as an allogeneic source owing to their low graft-*versus*-host disease (GvHD) (Liu et al., 2021). There are ongoing trials based on NK adoptive cell treatment (NCT04900454). One such elegant approach using chimeric receptor-expressing NK cells is being evaluated. In this, off-the-shelf IL-15 superagonist and GM-CSF neutralizing single-chain variable fragment (scFv) NK cells are being evaluated (Kundu et al., 2021). These dual cytokine-secreting cells will ensure the regulated activation of the adoptively transferred NK cells by the IL-15 superagonist and also handle the cytokine storm by neutralizing GM-CSF. Additionally, many more clinical trials are reported in ClinicalTrials.gov.in, which are evaluating the therapeutic potential of NK cells for COVID-19, the success of which will determine the application of this approach against any emerging SARS-CoV-2 VOCs.

## Monoclonal Antibody-Based Therapy

Monoclonal antibody-based therapy is currently considered the most successful treatment for COVID-19 besides the protection conferred by vaccines, reviewed by Zhou et al. (2021). A large repertoire of therapeutic monoclonal antibodies has been developed using various approaches, such as humanized animals, chimeric animals, phage display assay, and direct use of B cells from COVID-19 patients. In the next section, we will briefly discuss about the recent developments in the clinical application of therapeutic antibodies for COVID-19 treatment.

### Nasal Delivery of Antibody to Combat SARS-CoV-2

The delivery of neutralizing antibodies (IgG1 and isotypes) is usually carried out intravenously; however, these circulating antibodies lack efficient access to mucosal compartments, as is evident from a research carried out by DeFrancesco et al., where they have observed less antibody titer in the lungs as compared to the serum (DeFrancesco 2021). Delivering antibodies through the nasal route is an alternative to this approach and has an advantage for a pathogen causing respiratory tract infection such as SARS-CoV-2 because antibodies, if nebulized, can have greater access to the target cells compared to the intravenously delivered antibodies. Antibodies such as IgG and IgA1 have been reported to be nebulized through nasal inhalation (Vonarburg et al., 2019). The major challenge for antibody-based therapy is the resistance attained by newly emerging SARS-CoV-2 variants to different neutralizing IgG1 antibodies (Wibmer et al., 2021). To overcome such resistance encountered by IgG-based antibody therapeutics, an engineered IgM neutralizing antibody (IgM-14) was developed by Ku et al. (2021a). Compared to its parent counterpart (IgG-14), IgM-14 (engineered antibody) was observed to be >230-fold potent in neutralizing the SARS-CoV-2 pathogen. The resistant strains of SARS-CoV-2 and the VOCs, including the UK (B.1.1.7), Brazilian (P.1), and South African (B.1.351) strains, were shown to be neutralized by IgM-14 (Ku et al., 2021b).

### Antibody Cocktail Against SARS-CoV-2 Infection

The emergence of resistant strains due to antibody treatment interventions against pathogens such as respiratory syncytial virus (RSV) became the basis to developing an antibody cocktail approach against the SARS-CoV-2 pathogen (Simões et al., 2020). The antibody cocktail approach was observed to be beneficial against the rapid emergence of resistant mutations due to the single use of neutralizing antibody (Baum et al., 2020). Regeneron Pharmaceuticals developed an antibody cocktail with the trade name REGN-COV2 that contains a combination of two SARS-CoV-2 neutralizing antibodies: casirivimab (REGN10933) and imdevimab (REGN10987). The antibody cocktail containing two neutralizing and non-competing human IgG1 antibodies target the receptor binding domain of the SARS-CoV-2 spike protein, thus preventing viral entry *via* ACE2 (Baum et al., 2020; Hansen et al., 2020; Weinreich et al., 2021). A phase 1/3 clinical trial was conducted to determine the efficacy and safety of REGN-COV2, which involved hospitalized and asymptomatic COVID-19 patients. A total of 269 patients received REGN-COV2 and placebo, among which 90 patients were assigned to receive high-dose (8.0 g) REGN-COV2, 92 patients received a low dose (2.4 g), and 93 received placebo. Interim results showed low-grade and a few toxic effects of both low- and high-dose REGN-COV2. Adverse effects of special interest were reported in 2 out of 93 patients in the placebo group and 2 out of 176 patients in the REGN-COV2 group (Weinreich et al., 2021). Exogenous antibody treatment was better suited to those patients whose immune system had not yet been activated. However, patients who were already found serum-positive for neutralizing antibodies against SARS-CoV-2 were able to clear the virus efficiently. Based on these clinical findings, emergency use of this cocktail was granted in the 2021 for the treatment of COVID-19 in patients with mild to moderate symptoms. Similar antibody cocktails are being explored in various preclinical or clinical trials, and their success will determine the availability of this therapeutic modality against any emerging SARS-CoV-2 VOCs (Taylor et al., 2021).

## Vaccine Therapeutics

Vaccine is a substance, usually an inactivated pathogen or its component [protein or a messenger RNA (mRNA)], that, when introduced into the host, can elicit an immune response by imitating the pathogen’s infection process. This imitation of the infection lasts for a short duration until the body has developed long-term memory against the specific infection (Pulendran and Ahmed 2011). The aim of the vaccination is to prepare the body so that, in future, if there is an infection of the specific pathogen against which the vaccine was administered, memory cells will recognize and destroy the pathogen by releasing neutralizing antibodies. Vaccination, as a deliberate attempt at pathogen exposure, began in the lab of Louis Pasteur *via* his discovery of attenuated vaccine. He administered an aged culture of *Pasteurella multocida* to chickens that did not develop any disease-like symptoms; to his surprise, he observed that, on treating the same chickens with fresh culture of *P. multocida*, none of the subjects developed the disease (Pasteur, 1880). This observation led him to infer that aged culture had developed resistance against the pathogen in the chicken, which helped the subjects to fight against the infection of fresh bacterial culture. This observation led Pasteur to hypothesize that pathogens can be attenuated by any kind of environmental insults such as chemicals and high temperature, and the attenuated pathogen can bring immunity in healthy individuals against the disease-causing pathogen. Louis Pasteur confirmed his hypothesis further by working on rabies and anthrax (Pasteur, 1881). Due to the increased understanding regarding vaccine development, new endeavors of vaccine were brought into use, among which “antitoxin therapy,” also known as “serum therapy,” was an important discovery in the later part of the 19th century. Antitoxin preparations were first applied on animals, and the immunization studies on animals paved the way to applying this preparation in humans, which was utilized in 1931 (Moss 2020). The development of toxoid (inactivated toxin by formalin) took place in the early 1920s and is being used with certain minor modifications. Over the decades, knowledge regarding vaccine development has increased at an enormous pace, which paved the way to developing new vaccines within a short span of time and with better specificity and effectiveness against a particular pathogen.

### Types of Vaccines

Vaccines and vaccine development strategies have evolved with time, from traditional to new-generation vaccines. Traditional vaccines include whole pathogen vaccines, which can either be live-attenuated or inactivated vaccines. Live-attenuated vaccines consist of the pathogen with a highly reduced virulence (e.g., yellow fever/smallpox). On the other hand, inactivated vaccines are the chemically or thermally inactivated pathogens (Polack et al., 2020a). Developing live-attenuated or inactivated vaccines is an age-old and well-established strategy harboring rapid immunogenic property and providing immunological memory against specific pathogens. However, the drawback related to their use is the safety concern, which is due to live-attenuated vaccines having the potential to revert back to the virulent form and may infect immune-compromised subjects (Bazin 2003). Similarly, attenuated vaccines do not exhibit durable immunological memory and, hence, may not show long-term safety. Inactivation of a pathogen must neutralize the virulence without compromising its immunogenicity, as evident from previous research conducted to solve the mystery of why children in Washington, DC, developed disease after receiving the vaccine against RSV infection in the late 1960s. The research has highlighted the ineffective activation of immune response against the pathogen, as the RSV vaccine was not able to produce specific antibody response; rather, a hyper-immune response condition, also called enhanced respiratory disease (ERD), was elicited (Polack 2007). Advanced knowledge at the molecular level has highlighted an opportunity to identify and suppress the virulent genes of the pathogen to combat the problem of reversion of pathogenic virulence; however, safety concerns and short-term immunological memory persisted. Another approach is the subunit vaccine that consists of a part of the pathogen projected as an antigen to elicit immune response in the host organism. Adenoviral vectors are also being used to deliver some specific viral genes that can produce viral antigenic peptides in the host cells to elicit immune response (Kremer 2020b). Adenoviruses are non-fatal viruses that can infect humans and other organisms. Their exploitation as a vector for delivering viral antigens in host cells is a promising tool for the development of vaccines (Tatsis and Ertl 2004). Other vaccine types directly use the nucleic acids (mRNA and DNA) integrated with nano-technological approaches for delivery, as used by Moderna and Pfizer for COVID-19 vaccines, which will be discussed in the later section (Polack et al., 2020a; Corbett et al., 2020).

### General Mechanism of Vaccine Immunity

**FIGURE 1:**
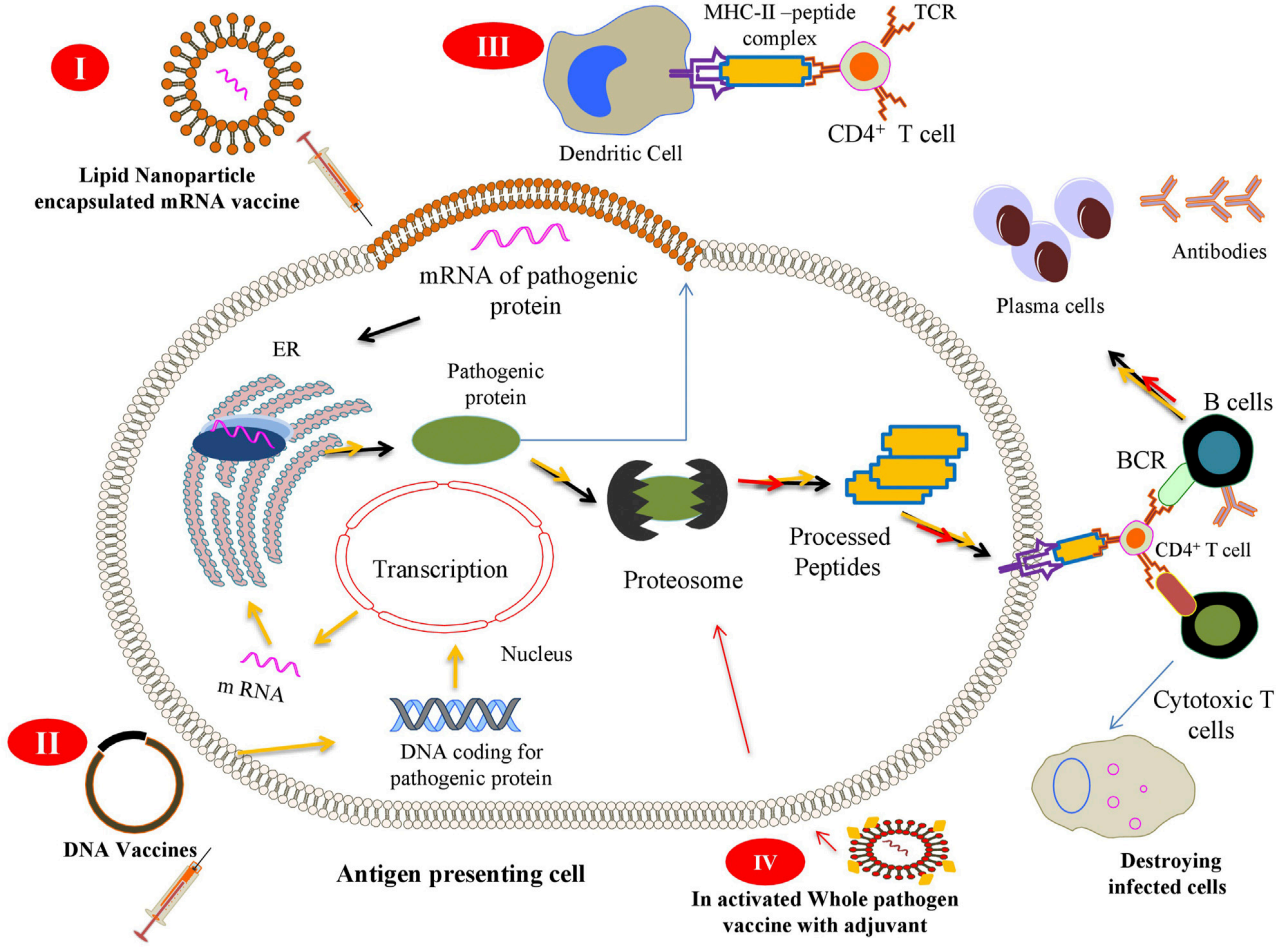
Scheme of the different vaccine strategies: (*I*) Messenger RNA (mRNA) vaccines contain mRNA encoding a pathogenic protein that acts as an immunogen to elicit cellular and humoral immune responses. The vaccine is injected as a lipid nanoparticle encapsulation that, once released inside (depicted by *black arrow*), the antigen-presenting cell (APC) is translated by cellular ribosomes and further presented on the surface of APCs. (*II*) DNA vaccines encoding a specific sequence for a pathogenic gene are captured by APCs that express and present the antigen to immune cells to elicit immune response (process depicted by the *yellow arrow*). (*III*) After expression of pathogenic proteins inside APCs, it may be secreted in the surrounding tissues (depicted by *cyan arrow*) where different other APCs can engulf, process, and present it to immune cells. (*IV*) Inactivated whole pathogen vaccines contain whole pathogens that have been subjected to heat or chemical inactivation when introduced into the body (depicted by *red arrow*) and stay avirulent, but pathogenic. This strategy is not able to bring an effective cellular immune response because of the inactivation of immunogenic proteins, thus needing an adjuvant (*yellow dots*) to bring about such a response in the cells.

The immune system reacts against challenges, whether it is from a pathogen or from an immunization, by activating its diverse components. Detection is carried out by the innate immune system by antigen-presenting cells (APCs). Within an APC, the antigen is processed to generate small protein fragments (peptides) that form a complex with major histocompatibility complex (MHC) molecules. This peptide–MHC complex is expressed on these cells, which further elicit an immune reaction by specifically engaging innate immune cells such as T or B cells. During immunization, either a whole pathogen or its component (protein) is introduced into the body in the form of a vaccine. This vaccine is detected as a non-self-entity by the APCs, which process it in the same way the pathogen is processed and subjected for antigen presentation to immune cells. Briefly, the peptide–MHC complex is expressed on the cell surface as an antigenic determinant (epitope), which activates T and B cells to neutralize the specific antigen. During this course, some T and B cells differentiate into memory cells and provide long-term memory against a particular pathogen (Clem 2011). Figure 1 shows a general scheme of a vaccine-mediated immune response after its delivery inside the human body.

The protective immunity against any viral infection has been attributed to the function of antibodies, especially neutralizing antibodies (Corti and Lanzavecchia 2013). These antibodies block the entry of viruses into the host cell, thus preventing infection. It has been the prime goal of vaccination to elicit adequate neutralizing antibodies. Different vaccines elicit different levels of serum antibodies serving as surrogates of protective immunity. These antibodies are measured using ELISA, lateral flow assay, hemagglutination assays, and live/pseudoviral-based neutralization assays (Amanat et al., 2020). The activation of immune cells against the vaccines depends upon the type of vaccine and its route of administration. Certain vaccines, like the cholera and typhoid fever vaccines that were designed to promote IgA response through oral administration, show lower efficacies. In contrast, parenterally administered vaccines elicit long-term immunity (Clemens et al., 2011).

The molecular players in vaccine-mediated immunity involve B cells, which are activated by the binding of antigenic determinants (from a pathogen or immunization) to B-cell receptors (BCRs) or by antigen-specific T helper cells. Pathogens like viruses have highly repetitive structures that can efficiently crosslink BCRs. However, soluble antigens lack such property and are usually ignored by B cells (Bachmann and Zinkernagel 1997). After the binding of antigen to the receptors, B cells interact with T helper cells and macrophages to differentiate in the clones of antibody-secreting cells, also called plasma cells and memory B cells. Plasma cells constitutively secrete antigen-specific antibodies, right after the pathogen attack. However, memory B cells are responsible for long-term antibody response (Amanna and Slifka 2011). Viruses usually bind to cell receptors; thus, the neutralizing antibody is directed against the viral protein that binds to the host cell receptor, thereby limiting the viral entry into the cells (Murin et al., 2019).

Long-term T-cell memory is a collaborative response of all T-cell populations against any cognate pathogen. CD4^+^ and CD8^+^ cells represent the specific cellular arm of adaptive immune response against viral infection or immunization. A subset of CD4^+^ T cells, also called T helper cells, recognizes processed antigens presented by APCs, especially dendritic cells (DCs). This process mounts a better immune response and memory compared to the immune response elicited by direct antigen binding by B cells (Kim et al., 2005). Detection of vaccine-associated signals by DCs conditions them to express molecules like interleukin 12 (IL-12), which further help T cells to proliferate and differentiate into various phenotypes (Alcaide et al., 2021).

## COVID-19 Vaccines

The COVID-19 pandemic has put pharmaceutical companies and laboratories in a race to develop an effective and rapid COVID-19 vaccine. Both traditional as well as modern vaccine development strategies have been employed to develop an effective and safe vaccine for COVID-19. This becomes imperative when there is no suitable drug available in the market, owing to the novelty of the pathogen causing COVID-19. As of October 2021, around 194 vaccine candidates have been in the preclinical phase and 126 vaccine candidates in the advanced clinical phases (https://www.who.int/publications/m/item/draft-landscape-of-covid-19-candidate-vaccines). Due to the availability of prior knowledge regarding the pathogenesis and immune responses of SARS-CoV and Middle East respiratory syndrome-related coronavirus (MERS CoV), the vaccine development against SARS CoV-2 started at a remarkable pace (Folegatti et al., 2020a). Early pieces of evidence have suggested that neutralizing antibodies against the spike protein might be an important hallmark for immunity against SARS-CoV-2 infection (Wajnberg et al., 2020). These reports thus formed the basis for developing the COVID-19 vaccine, many of which have successfully passed clinical trials and are currently available in the market, as reviewed recently (Kyriakidis et al., 2021). Most COVID-19 vaccines that obtained an emergency approval for their therapeutic use have been recommended for the population beyond the age of 18 years, and in most countries, these vaccines are yet to be tested in children below 18 years. Certain vaccines that are in the early stage of drug trials are reported to be intranasally administered, including ChAdOx1-S, AdCOVID, BBV154, and COVI-VAC, which may become the leading vaccines to be used for those below 18 years old (Lund and Randall 2021). Such vaccines have been used in the past to treat respiratory pathogens in children (Mohn et al., 2018). There are challenges that can be encountered in the future regarding the efficacy of the COVID-19 vaccines, as the SARS-CoV-2 pathogen is rapidly evolving owing to its RNA genome (MacLean et al., 2020). Thus, many COVID-19 vaccines are being tested for their efficacy against the different VOCs currently circulating in places where drug trials are being carried out. Table 1 summarizes certain important and approved vaccines and their status of efficacy and the drug trial stages. In the next section, we will discuss the basic biological features and clinical information of the candidate vaccines currently approved or undergoing the final phase of clinical trials for COVID-19.

**TABLE 1:** Trial stages of some important COVID-19 vaccines and their efficacy with respect to different SARS-CoV-2 variants

S/No.	Vaccine	Phase of drug trial	Trial number	Effectiveness against VOCs	Reference
1	Pfizer	Phase III trial completed	NCT04368728	B.1.351, P.1, B.1.427/B.1.419, P.2, and B.1.526	Polack et al. (2020b)
2	Moderna	Under phase III drug trial	NCT04470427	B.1.427/B.1.429 and B.1.526	Baden et al. (2021)
3	CoronaVac	Under phase III drug trial	NCT04582344	P.1 and P.2	Tanriover et al. (2021)
4	BBIBP-CorV	Under phase III drug trial	NCT04984408	No information	Al et al. (2021)
5	BBV152	Under phase III drug trial	NCT04641481	B.1.617.2 and B.1.617.1	Ella, Reddy, et al. (2021)
6	Sputnik	Under phase III drug trial	NCT04656613	No information	Callaway (2020)
7	Ad26.CoV2.S	Under phase III drug trial	NCT04505722	B.1.351, P.1, B.1.427/B.1.429, P.2, B.1.526, and C.37	Sadoff et al. (2021)
8	Covisheild	Under phase III drug trial	NCT04324606, NCT04400838 NCT04444674	B.1.1.7, B.1.351, P.1, B.1.427/B.1.429, P.2, B.1.526, and C.37	Voysey et al. (2021)
9	NVX-CoV2373	Under phase III drug trial	NCT04611802	B.1.1.7, B.1.351, B.1.427/B.1.429, and B.1.526	Heath et al. (2021b)

VOCs, *variants of concern*

### mRNA-Based COVID-19 Vaccines

*Pfizer/BioNTech mRNA Vaccine*: Tozinameran, code named BNT162b2, is more commonly known as Pfizer/BioNTech COVID-19 vaccine. It is being sold in the market with the trade name Comirnaty. This vaccine is based on mRNA (encoding the full-length SARS-CoV-2 spike protein) encapsulated in a lipid nanoparticle formulation (Polack et al., 2020a). The first country to authorize Tozinameran for emergency use was the UK. This vaccine is administered as a two-dose regimen (30 μg each), given 21 days apart. The dosage regimen has been observed to elicit optimal immune response in the form of neutralizing antibodies and effective T-cell responses after 28 days of administration (Mahase 2020). A local and short-term reactogenicity profile was observed in the second phase of drug trials at the injection site. An early trial report indicated that, initially, a total of 44,820 people (with mean age of ≥16 years) were screened, among which 43,548 underwent randomization at different sites round the globe. In the phase 2/3 trial, 43,448 participants received injection, among which 21,728 were administered placebo and 21,720 participants received BNT162b2 (NCT04368728). During the follow-up, around 27% of BNT162b2 recipients and 12% of placebo recipients reported adverse but manageable side effects. Lymphadenopathy was reported in 0.3% of vaccine recipients and <0.1% placebo recipients. In terms of efficacy, it was observed that BNT162b2 had an impressive 95.0% efficacy in the early trial results (Polack et al., 2020a). However, the major limitation of this vaccine is the need for storage at lower temperatures ranging from −80°C to −60°C, which is challenging for most of the developing countries. Drug trial 2/3, which was carried out to assess the safety and immunogenicity of 30 μg BNT162b2 (given 21 days apart) among participants 16 years of age or older, found the vaccine to be safe with an efficacy of 95% (Polack et al., 2020b). This is the first vaccine to obtain stringent regulatory authorization and clearance for its emergency and regular use in the United States. It is fully authorized in five countries and has an emergency use authorization in around 108 countries.

*mRNA-1273 vaccine*: The joint collaboration of Moderna, Biomedical Advanced Research and Development Authority (BARDA), and National Institute of Allergy and Infectious Diseases (NIAID) led to the development of another mRNA-based SARS-CoV-2 vaccine, more commonly called Moderna COVID-19 vaccine and code named mRNA-1273. It is sold in the market under the brand name Spikevax. This vaccine also contains an mRNA encoding the SARS-CoV-2 spike glycoprotein enclosed in a lipid nanoparticle encapsulation (Corbett et al., 2020). This vaccine is administered intramuscularly in two doses of 0.5 ml within a gap of 2 weeks.

In the first phase of the drug trial, 45 participants were enrolled to receive the mRNA-1273 vaccine, which was initially given in three different concentrations (25, 100, and 250 μg) in 15 participants each (NCT04283461). The vaccine was provided in the form of a sterile liquid for injection at a concentration of 0.5 mg/ml. Two consecutive doses of each concentration were given with a gap of 28 days. No participant developed any serious complication immediately after the first vaccination. In the second vaccination process, no participant reported any adverse effect in the 25-μg group; however, 40% of the participants in the 100-μg group and 57% of the participants given 250 μg reported minor complications such as fever, mild fatigue, chills, and pain at the injection site. The published drug trial reports indicated that mRNA-1273 was effective enough to develop immune response *via* virus-neutralizing antibodies in the recipients (Jackson and ColerMcCullough, 2020). Rapid seroconversion was observed 2 weeks after the administration of the first vaccine dose. However, pseudoviral neutralization was found to be low in the first dose, which signifies the need for a second dose of this vaccine. Drug trial III was conducted at 99 centers across the United States, and 30,420 volunteers took part in the trial. mRNA-1273 showed 94.1% efficacy in preventing COVID-19 illness **(**NCT04470427) (Baden et al., 2021). Unlike BNT162b2, mRNA-1273 does not need to be stored at −80°C, but instead can be stored between −25 and −15°C. However, the thawed vaccine cannot be refrozen and should be stored between 2°C and 8°C for use within a short time frame. This vaccine obtained emergency use authorization by the Food and Drug Administration (FDA) on December 18, 2020. Since then, the health ministries of various other countries, including the European Union, United States, and Canada, have also approved its emergency use.

#### Advantages and Limitations

mRNA vaccines are developed in a microbial-free environment and are also non-infectious molecules. These features discriminate them from the other vaccine regimens such as inactivated viral vaccines, live attenuated viral vaccines, and vector-based vaccines in terms of safety and efficacy. mRNA vaccines are free from the issue of anti-vector immunity and are inexpensive compared to the other vaccines. As the mRNA is restricted to the cytosolic region, there is no chance of integration within the host genome, thus ensuring that cells do not get transformed (Pardi et al., 2018). Their adaptability and simple design provide rapid and easy production for any emerging strain, which thus makes mRNA-based vaccines more attractive.

Certain limitations may be associated with their use, as the formulation of modified mRNA-based vaccines is being employed for the first time on large populations; thus, it is too early to discuss their long-term safety concerns and complications. Specifically, the nanoparticle conjugates used to deliver mRNA into the cells need further investigation. According to a few studies, mRNA-based vaccines formulated with nanoparticles may induce type I interferon response associated with potential autoimmunity and inflammation, which warrants further study (Zhang et al., 2019).

### Inactivated Whole Pathogen Vaccines

*CoronaVac*: The vaccine candidate formerly known as PiCoVacc is an inactivated vaccine (CNO2 strain of virus) developed against SARS-CoV-2 by the China-based biotechnology company Sinovac Biotech. This vaccine is being sold under the trade name “CoronaVac.” The vaccine was derived from African green monkey kidney cells (Vero cells) inoculated with SARS-CoV-2. The virus was inactivated by β-propiolactone and adsorbed onto aluminum hydroxide. The vaccine is administered in two doses with a gap of 14 days and has obtained an emergency use approval by the Chinese government for high-risk groups and frontline COVID-19 warriors. Doses of 3 and 6 μg of the vaccine are administered intramuscularly as per the dosing schedule (Zhang et al., 2020). Some participants have reported mild adverse effects, and the incidence of these reactions was dose-independent, signifying that there is no dose-related safety concern for this vaccine (NCT04352608) (Gao et al., 2020). This inactivated vaccine candidate is under phase III trial, currently ongoing in Brazil, Chile, Turkey, and Indonesia. On January 12, 2021, Brazil announced an efficacy of 50.38% from a study on 12,508 participants, whereas the trial reports from Turkey showed 91.25% efficacy. However, the data from Turkey were based on only 29 COVID-19 cases among 1,322 trial participants; thus, further results from enrolled participants will provide more explicit data on the efficacy of this vaccine (Mallapaty 2021).

*BBIBP-CorV*: BBIBP-CorV (Biotech Group and Beijing Institute of Biological Products—Coronavirus Vaccine) is another promising vaccine candidate developed by Beijing Institute of Biological Products, China. It is also a whole inactivated virus vaccine developed using the HBO2 strain of SARS-CoV-2 derived from Vero cells. Inactivation was carried out by treating the viral supernatant with β-propiolactone. This vaccine can be stored and transported at refrigeration temperatures (2–8°C). An early trial report conducted at Shangqiu City Liangyuan District Center for Disease Control and Prevention, Henan Province, China, has suggested that BBIP-CorV is an effective vaccine in all the age groups enrolled in the study. Vaccine/placebo recipients were separated randomly into two age groups: 18–59 and 60 years. The vaccine was administered at a two-dose schedule (2, 4, or 8 μg) with a gap of 4 weeks. The same immunogenicity profile was observed for the vaccine in both age groups. The anti-SARS-CoV-2 neutralizing antibody titer was higher at day 42 in the age group 18–59 years. BBIBP-CorV was also found to induce sufficient amount of immune response in participants 60 years and older (Xia et al., 2021). Adverse reactions were reported within 7 days of vaccine inoculation in 29% of the participants; however, these adverse reactions were found to be mild. BBIBP-CorV is currently tested in trial III, which started on October 1, 2021 with the estimated date of completion on September 30, 2024. The study will involve volunteers aged 18 years (NCT04984408).

*BBV152*: BBV152, with the trade name “Covaxin,” is an inactivated whole pathogen vaccine against SARS-CoV-2. It was developed by Bharat Biotech in collaboration with the Indian Council of Medical Research (ICMR). Bharat Biotech obtained an approval to conduct its early phase drug trials on June 20, 2020. BBV152 is a whole SARS-CoV-2 virus inactivated by β-propiolactone and developed from a strain of SARS-CoV**-**2 (NIV-2020**-**770) isolated and sequenced from the National Institute of Virology of ICMR, Pune, and provided to Bharat Biotech for vaccine development. NIV-2020-770 contains a D614G mutation at its spike protein. The whole pathogen vaccines were formulated with alum, which is reportedly less likely to induce cell-mediated immunity. However, considering the desirability of cell-mediated immune response, an imidazoquinoline molecule, a Toll-like receptor (TLR7/8) agonist was used to induce cell-mediated immune response (Philbin et al., 2012). To deliver the vaccine antigen directly to the lymph nodes and avoid direct diffusion into systemic circulation, imidazoquinoline chemisorbed on alum (Algel-IMDG) was designed. Preclinical trials conducted in non-human primates showed enough viral clearance in vaccinated subjects using this strategy (Yadav et al. 2020).

Between July 13 and 30, 2020, a total of 375 participants took part in the first drug trial, among which 100 participants were assigned randomly to receive either one of the three formulations of the vaccine (3 μg Algel-IMDG, 6 μg Algel-IMDG, or 6 μg Algel) and 75 comprised the control group given Algel only (without antigen; NCT04471519). Two doses of the vaccine were administered intramuscularly with a gap of 14 days. Common adverse effects after vaccine administration were injection site pain, headache, vomiting, fever, and nausea. All the adverse effects were mild or moderate and were frequent after the first dose only. The tolerable safety outcomes and the enhanced immune response led both vaccine formulations (3 and 6 μg) to enter phase II of immunogenicity trials (EllaRachers et al., 2021a). This vaccine can be stored at 2–8°C, making it compatible to the cold chain requirements. The Lancet reported interim findings of the safety and immunogenicity of BBV152 at three different formulations and the control group Algel (EllaRachers et al., 2021a). Phase III of the drug trials was conducted between November 16, 2020 and January 7, 2021. In this trial 25,798 participants took part, among which 12,221 received the vaccine (two doses) and 12,198 participants received placebo (NCT04641481). BBV152 was found to be 77.8% efficient against the Wuhan strain of SARS-CoV-2; however, efficacy against a VOC (B.1.617.2, Delta) was observed to be only 65.2% (EllaRachers et al., 2021b). This vaccine obtained emergency use approval in India on January 2, 2021 (Mohapatra and Mishra 2021).

#### Advantages and Limitations

In an inactivated vaccine, the pathogen loses the infectivity potential faster than in other vaccine types, but the antigenicity profile is maintained for longer. Thus, these vaccines are considered relatively safer than the vaccines based on live pathogens. Furthermore, the immunogenicity of inactivated viruses is lower and needs a booster dose to be administered, which usually increases the cost. The use of adjuvants can cause unwanted inflammatory responses (Gendon 2004). In the past, inactivated vaccines have been known to induce enhanced disease among the recipients. In fact, the trials for formalin-inactivated RSV had disastrous outcomes, as 80% of the vaccine recipients were hospitalized and two recipients died as a consequence of enhanced disease (Kapikian et al., 1969). However, considering the early success of the use of these vaccines for COVID-19, similar approaches may be used for developing vaccines against the emerging VOCs, if needed.

### Adenovirus Vector-Based Vaccines

*Russian Sputnik V SARS-CoV-2 Vaccine*: Gam-COVID-Vac, with the trade name Sputnik V was the first vaccine against COVID-19 announced by the Russian Government on August 11, 2020. The vaccine is based on two human non-replicating adenoviral vectors—recombinant adenovirus 26 (rAd26-S) and recombinant adenovirus 5 (rAd5-S)—both of them carrying the gene related to the spike protein of SARS-CoV-2. It was developed by Gamaleya National Center of Epidemiology and Microbiology, Moscow, Russia (Burki 2020). This approach has been used in the past to develop a vaccine against Ebola (Dolzhikova et al., 2017). The vaccine was made in two formulations, frozen and lyophilized, and is administered intramuscularly. Both formulations require different storage temperatures: −18°C for the frozen formulation and 2–8°C for the lyophilized one. To assess the safety and immunogenicity of both vaccine formulations, a non-randomized study was carried out at Burdenko Hospital and Sechenov University, Moscow, Russia, which included 120 healthy participants aged 18–60 years (NCT04530396). A full dose contained 10^11^ viral particles, and every participant under study was given a full dose of the vaccine. The volume of the frozen vaccine was 0.5 ml and that of the lyophilized vaccine needed to be reconstituted in 1 ml of sterile water (Logunov et al., 2020). The findings of the phase 1/2 drug trial, as reported by The Lancet, have provided feasible results (Logunov et al., 2020). The vaccine was found safe and well tolerated, with adequate cellular and humoral immune response in healthy volunteers. On day 14 post-administration of rAd26-S and rAd5-S, SARS-CoV-2 receptor-binding domain (RBD)-specific IgGs (neutralizing antibodies) were detected in 88.9% and 84.2% of participants, respectively. The adverse effects reported post-vaccine administration included hyperthermia, headache, muscle and joint pain, and pain at the injection site. No serious adverse effects were reported during the study (Logunov et al., 2020). The neutralizing antibody titer was lower compared to that of mRNA-based vaccines (Jackson ColerMcCullough, 2020). Based on the successful clinical trial results, Sputnik V was approved in more than 60 countries for COVID-19 (Lawton 2021).

*Ad26.COV2.S vaccine by Johnson & Johnson*: Ad26.COV2.S is a vaccine candidate developed by Janssen Pharmaceuticals of Johnson & Johnson in collaboration with Leiden University Medical Center, Netherlands. It is a replication-defective vaccine expressing the full-length spike glycoprotein of SARS-CoV-2 (Poland et al., 2020). Ad26.COV2.S is in phase 3 trial involving 60,000 participants aged 18 years or older. Induction of strong neutralizing antibodies was observed after a single immunization (1 × 10^11^ viral particles introduced intramuscularly) of this adenovirus-based vaccine in rhesus macaques (Mercado et al. 2020). This vaccine can be stored at 2–8°C. Drug trial 1/2a was conducted among healthy adults aged 18–55 years (cohort 1) and 65 years and above (cohort 3). The vaccine was administered in a single- or two-dose regimen (5 × 10^10^ or 1 × 10^11^ viral particles per vaccination) within an interval of 56 days. Interim results indicated that there was a paramount development of S-binding antibody titer in 90% of the participants regardless of age and vaccine dose (NCT04436276). Detectable neutralizing antibodies were observed in 98% of the participants 29 days after vaccination (Sadoff et al., 2020). Phase 3 trial reports involving around 40,000 participants indicated that a single dose of the Ad26.COV2.S vaccine provides protection against symptomatic and asymptomatic SARS-CoV-2 infections (NCT04505722). Vaccine efficacy was found to be higher against severe to critical COVID-19, around 76.7% for onset at >14 days (Sadoff et al., 2021). The FDA issued emergency use authorization of the Johnson & Johnson COVID-19 vaccine on February 27, 2021.

*ChAdOx1 nCoV-19*: ChAdOx1 nCoV-19 is a chimpanzee adenovirus-based SARS-CoV-2 vaccine developed by Oxford University (ChAdOx1) and is in the market under the trade name “Covishield.” It is a replication-deficient adenoviral vector carrying the SARS-CoV-2 surface glycoprotein antigen gene (spike protein). This strategy has already been tested in the past for other pathogens such as MERS CoV (Jia et al., 2019). Since the use of human adenoviral vaccines may have a reduced immunogenicity profile due to preexisting immunity to human adenoviruses, using chimpanzee-derived vectors was thus a better strategy of developing a vaccine with better immunogenicity against the SARS-CoV-2 antigen owing to the low seroprevalence of antibodies against chimpanzee-derived adenoviral vectors (Ramasamy et al., 2020). As per previous studies, the prevalence of antibodies against chimpanzee adenoviruses was found to be 0% in tested individuals among the UK population and 9% in the Gambia population, which provided an insight into the safe usage of adenoviral vectors for vaccine delivery (Morris et al., 2016).

Phase 1/2 trial reports published in The Lancet indicated acceptable profiles of immunogenicity and safety related to the ChAdOx1 vaccine (Folegatti et al., 2020a). The report was published from a phase 1/2 trial conducted in the UK on 1,077 participants (18–55 years old) who have received the vaccine (NCT04324606) (Folegatti et al., 2020b). In the phase 2 trial, older participants (>65 years) received the vaccine, and a 62% efficacy was observed in the two full-dose regimen administered with a gap of 1 month (Ramasamy et al., 2020). However, better efficacy was observed when a lower dose of the vaccine was used followed by a full dose. No COVID-19-related hospitalization was reported in the ChAdOx1 vaccine recipients, while 10 volunteers from the control (placebo recipients) group were hospitalized due to unrelated complications (Knoll and Wonodi 2020). Considerable binding and neutralizing antibody induction were observed after the administration of the second dose. This vaccine has been observed to be better tolerated in older participants with uniform immunological profile in all age groups after administration of a booster dose, thus becoming a ray of hope for the elderly population who are at greater risk of severe COVID-19 (Folegattiet al., 2020b). Drug trial 2/3 carried out between May 30 and August 8, 2020 has highlighted the safety and efficacy of ChAdOx1 nCoV-19 in different age groups, especially in the age group 70 years and above **(** NCT04400838 **)**. The vaccine was administered either as a single dose or as a two-dose regimen at a low dose (2.2 × 10^10^ viral particles) or a standard dose (3.5–6.5  10^10^ viral particles). Multiplex immunoassay was used to determine the total IgG levels against the trimeric spike protein and RBD. At both dose levels, the anti-spike IgG response was found to be decreased with increasing age. However, participants who received a booster dose had similar antibody titers regardless of age or vaccine dose, and the titer was higher compared to that in participants who did not receive a booster dose. A similar trend was observed with the anti-RBD IgG (Ramasamy et al., 2020). In India, the vaccine is sold under the brand name Covishield and was first approved on December 30, 2020.

#### Advantages and Limitations

Adenoviral-based vectors are considered relatively safe due to their low immunogenicity in humans. These vectors are efficient in inducing antibody- and cell-mediated immune responses, probably due to the efficient expression of the encoded gene of interest. Long-term immunity requires prime booster doses. Thus, usually, a two-dose regimen is followed for adenoviral vectors to induce memory T- and B-cell immune responses (Geisbert et al., 2011).

Human adenoviruses have long since been known to mankind; thus, preexisting antibodies against these vectors can be a drawback for vaccine development. Certain rare but grave adverse reactions after administration of human adenoviral-based vaccines have been observed, which could be the cause of the preexisting anti-adenoviral immunity in the subjects who have been previously infected by multiple adenoviruses (Kremer 2020a). To induce a stringent amount of immune response and memory cells, adenovirus-based vaccines need booster doses, which may increase the cost of their preparation. Thus, to overcome these limitations, it is imperative to use adenovirus vectors from other species, such as the chimpanzee adenovirus used in ChAdOx1. The long-term side effects of these vaccines will be monitored over the years.

### Protein Subunit Vaccines

*NVX-CoV2373 vaccine*: Novavax developed a recombinant SARS-CoV-2 (rSARS-CoV-2) nanoparticle vaccine against the S-protein also known as NVX-CoV2373. The vaccine was manufactured at Emergent BioSolutions and constructed from the full-length, wild-type SARS-CoV-2 trimeric S-protein with the Matrix-M1 adjuvant. Optimization was done in the already established baculovirus *Spedoptera frugiperda* (Sf9) insect cell expression system (Tian et al., 2020). The rSARS-CoV-2 vaccine has been modified, wherein certain mutations such as 682QQAQ685 at the S1/S2 cleavage site of the S-protein confer protease resistance to the vaccine and at the same time two proline substitutions in residues K986P and V987P in the S2 subunit help in stabilizing the construct in a profusion conformation. This strategy makes NVX-CoV2373 resistant to proteolytic cleavage and allows it to bind to hACE2 receptors with high affinity. The Matrix-M1 adjuvant is a saponin-based adjuvant, a product of Novavax (Wrapp et al., 2020). Trial 1/2 was initiated to evaluate the safety and immunogenicity of the NVX-CoV2373 vaccine given in 5 and 25 μg doses with or without the adjuvant (NCT04368988). The trial started on May 26, 2020 on 134 study participants, among which 83 participants received the vaccine with adjuvant, 25 without adjuvant, and 23 participants were assigned to receive placebo (0.9% normal saline). Three participants served as a backup during the trial run. Mild side effects were seen, and a few cases of adverse events in different groups were observed, which became stable after a few days (less than 7 days). Thus, overall, no severe adverse events were reported in the early trial results. The primary immunogenicity profile of the 5- and 25-μg doses of the vaccine plus adjuvant showed acceptable neutralizing antibody response correlating with anti-spike IgG (Keech et al., 2020). Both vaccine and adjuvant can be stored at 2–8°C.

Furthermore, in a phase 3 trial, 14,039 participants (7,020 in the vaccine group and 7,019 in the placebo group) were enrolled as per protocol and included for population efficacy (NCT04583995). A two-dose regimen of the NVX-CoV2373 vaccine was given 21 days apart, and an efficacy of 89.7% was observed (Heath et al., 2021a). There were no hospitalizations or deaths among the 10 cases in the vaccine group: however, five cases of severe infection were reported among 96 in the placebo group. It was also worth observing that this vaccine was effective against variant B.1.1.7 and the non-B.1.1.7 variants, and the efficacy rates were observed to be 86.3% and 96.4%, respectively, using a *post-hoc* analysis (Heath et al., 2021a). Shinde et al. carried out a study on the efficacy of NVX-CoV2373 against the B.1.351 variant of SARS-CoV-2. This was a phase 2-a trial that started in South Africa, where 6,324 participants received a single dose of vaccine/placebo. The recruited participants were either HIV-negative or medically stable HIV-positive. The vaccine was found to be efficacious in preventing COVID-19, with a higher efficacy rate among HIV-negative participants (NCT04533399) (Shinde et al., 2021).

#### Advantages and Limitations

As the vaccine is devoid of any infectious particle, it is thus safe for use. The benefit of using an adjuvant was clear in terms of the high magnitude of antibody- and cell-mediated immune responses compared to the antigenicity profile of the vaccine without adjuvant. This vaccine can be stored at 2–8°C, thus mitigating cold chain storage and transport demands. The vaccines contain purified antigenic peptides to elicit immune response, thus avoiding the use of live components of the pathogen.

The limitation, as with all other vaccines, is that it needs booster doses to provide long-term immune response against the pathogen. The immune response remains low without the adjuvant; thus, the need of an adjuvant increases the cost of vaccine formulation. Since the isolated peptide is used to design the vaccine, the other limitations related to this technique is that the isolated protein, if denatured, may bind to nonspecific antibodies.

## Future Perspectives

After the onset of the COVID-19 pandemic, an immediate need for treatment arose to limit the spread of the disease. Initially, repurposed antiviral drugs and other anti-inflammatory treatment regimens were tested. Antiviral drugs such as remdesivir had a significant effect of minimizing the hospitalization in a subset of patients, but this drug also fell short of expectations (Hoek and Sarahanne, 2021; Masoomikarimi et al., 2021). Similarly, CPT showed promising results in earlier clinical trials, but this therapy was also limited due to technical difficulties and safety concerns. However, antibody-based therapies presented a promising approach in dealing with SARS-CoV-2 infection, and many therapeutic antibodies were approved for the treatment of patients with mild and moderate symptoms (Ning et al., 2021). Besides the existing approaches for developing therapeutic antibodies, several engineered antibody-based therapies, including antibody cocktails and nasal sprays, showed promising clinical outcomes (Baum et al., 2020). These short-term protective therapeutic modalities may also work against the emerging SARS-CoV-2 VOCs, but a long-term and sustainable treatment is what would change the COVID-19 infection landscape. In this direction, vaccines will remain the mainstay therapeutic modalities for COVID-19, which has already documented successful and lasting protective immunity. Modern vaccines such as those based on mRNAs designate a new era in vaccinology as the approach is being used against infectious diseases, and they appear ideal for use against any emerging SARS-CoV-2 variants and non-pathogenic diseases such as cancer (Pardi et al., 2018). Notably, the pan-COVID-19 vaccine (Saunders et al., 2021), which is in the making, holds an enormous promise to target multiple and any new variants of coronaviruses—a hope for the future.
